# The Hospital Conference, 1908

**Published:** 1908-04-11

**Authors:** 


### The Hospital

A Journal of

The Medical Sciences and Hospital Administration.

New Series. No. 56, Vol. III. [No. 1128, Vol. XLIV.] SATURDAY, APRIL 11, 1908.

### The Hospitals Conference, 1908.

As we pointed out would be the case in our issue of January 25, the proceedings at this Conference last week, though they resulted in the passing of one or two resolutions embodying important principles, have in practice proved mainly abortive. It was hopeless to expect that practical or useful purposes will be fulfilled by such a Conference until its organisation is managed as a separate undertaking, with properly constituted officers under a working committee representing the hospitals and the best hospital opinion. The so-called United Kingdom Hospitals Conference was throttled by the circumstance that it is in the hands of the Joint Hospitals Committee of the British Medical Association, that it is without funds, that it forms but a subsidiary portion of a mass of work undertaken by the British Medical Association, and that in consequence the preliminary work in connection with it was left untouched for more than twelve months after the meeting in 1906. It is not surprising therefore that the discussions which followed the subjects introduced at the Conference should be disappointing.

Put concretely the object which underlies the whole movement is rebellion on the part of the general practitioners against the present system of out-patient relief granted by the hospitals. We have the fullest sympathy with the demand of the general practitioner for reform in regard to out-patient relief, but the curious fact remains that several resolutions passed last week, and the contentions of several speakers, as will be seen from the full report we give elsewhere of the proceedings, embody principles which must adversely affect the income of the majority of the working members of the medical profession everywhere. If such developments are to be permitted they must speedily reduce the number of entries at the medical schools, for it may soon prove impossible for the general practitioner to live by his profession.

It is matter for regret that the gathering should have been denominated The United Kingdom Hospitals Conference at all. In fact the hospitals of the United Kingdom, and especially the metropolitan hospitals, were practically unrepresented in any effective sense. Less than one hundred people were present at the meetings, and representatives of the principal hospitals, that is the active spirits who are responsible for the management of the hospitals, were conspicuous by their absence. With a few exceptions none of the representative managers of the principal hospitals took any part in the proceedings, and it is matter for regret that the lay press should have accepted the misleading title given to the meetings last week, under the impression that this Conference did in fact represent the interests of the hospitals, and that it comprised a representative gathering of hospital managers, supporters and honorary medical officers who collectively are responsible for the maintenance and administration of our hospitals as they exist to-day. Anything more misleading than this impression it would be impossible to imagine, for, as we have pointed out, with a very few exceptions the responsible representatives of these various sections of our hospital system were conspicuous by their absence.

It is matter for serious regret in these circumstances, though not at all surprising, that a false impression should have been created in the minds of the press and the public, who have been led to conclude that grave abuses requiring immediate remedy almost everywhere prevail in the administration of the voluntary hospitals of this country. Anything more unfortunate and more widely divorced from the truth than such an impression it would be difficult to imagine. Never in the whole history of our hospitals has the spirit of reform been more actively at work in the direction of improved administration than during the last ten years, and the results which have been achieved have placed the largest and best hospitals of this country in a position of high administrative efficiency which has deservedly won the admiration of foreigners who have an intimate knowledge of hospital affairs. It is certainly time that the hospital managers should use their influence to bring immediate pressure to bear upon the members of the Committee appointed on the 2nd instant, with the object of securing that the next Conference, if it is to be held at all, shall be so organised as to make it representative of the best interests of the hospitals and of all who are intimately associated with their efficient administration.

The effect of last week's Conference cannot be beneficial to the hospitals owing to the misapprehensions which have arisen in regard to its representative character, and the sooner the hospitals take action the better, with a view to protecting their interests and making the next Conference what it ought to be and must be, if it is to do good and not harm to our present hospital system. Instead of a gathering consisting for the most part of men who have ample leisure, rather than great hospital experience, we need a Conference of the active workers attached to our hospital system, who shall be representative of every interest and every opinion which is worthy of consideration. Let it be the business of each great hospital to bring influence to bear upon the existing Conference Committee without delay. Either the present machinery should be destroyed altogether, or by prompt action care should be taken to secure that any Hospital Conference which may be held in 1909 shall in fact prove to be representative, practical, and in every respect as useful as possible to the best interests of our present hospital system.

